# Non-invasive anemia detection from conjunctiva and sclera images using vision transformer with attention map explainability

**DOI:** 10.1038/s41598-025-32343-w

**Published:** 2025-12-19

**Authors:** Oscar Ramos-Soto, Jorge Ramos-Frutos, Ezequiel Perez-Zarate, Diego Oliva, Seyed Jalaleddin Mousavirad, Sandra E. Balderas-Mata

**Affiliations:** 1 Depto. de Ingeniería Electro-fotónica, Universidad de Guadalajara, Guadalajara, Jalisco 44430 México; 2 Posgrados, Centro de Innovación Aplicada en Tecnologías Competitivas, León, Guanajuato 37545 México; 3 Depto. Ingeniería Industrial, Tecnológico Nacional de México/Instituto Tecnológico de Jiquilpan, Jiquilpan, Michoacán 59514 México; 4 Coordinación General Académica y de Innovación, Universidad de Guadalajara, Guadalajara, Jalisco 44100 México; 5 Department of Computer and Electrical Engineering, Mid Sweden University, Sundsvall, Sweden

**Keywords:** Iron-deficiency anemia, Vision transformer, Non-invasive diagnosis, Explainability, Conjunctiva and sclera images, Biomedical engineering, Electrical and electronic engineering

## Abstract

Iron-deficiency anemia, a prevalent global health issue, traditionally requires invasive procedures for accurate diagnosis, such as a blood sample for measuring hemoglobin (Hgb) concentration. Nevertheless, this marker can be visually assessed by observing external anatomical elements, such as the eye’s conjunctiva and sclera. These regions often appear paler in anemic individuals, providing a visual sign of potential anemia. In this work, a non-invasive approach for anemia detection utilizing sclera-conjunctival images is presented. Using the Vision Transformer (ViT) model with a transfer learning approach, robust classification of anemia/no anemia is achieved. This methodology not only focuses on classification accuracy but also incorporates an explainability technique to provide visual insights into the decision-making process of the model. Experimental results demonstrated high accuracy, where an overall accuracy of 98.47% is achieved. The ViT model’s performance is compared against established machine learning and deep learning algorithms to evaluate its effectiveness in anemia detection. The analysis of the results indicates that the ViT model, with its ability to focus on relevant image features when analyzing the explainability results, offers a promising alternative for anemia detection, potentially reducing the need for invasive diagnostic procedures.

## Introduction

Iron-deficiency anemia is one of the most prevalent nutritional disorders worldwide, affecting a substantial portion of the global population^1^. It typically arises from inadequate dietary iron intake, chronic blood loss, or increased iron demands, resulting in a reduced number of red blood cells or lower hemoglobin levels^2^. This condition decreases the blood’s ability to transport oxygen^3^, leading to symptoms like fatigue^4^, weakness^5^, and cognitive impairments^6^, all of which can significantly diminish the quality of life. In severe cases, iron-deficiency anemia can lead to serious health complications, including heart issues and pregnancy complications^7,8^.

Traditionally, anemia is diagnosed through blood tests that measure Hgb levels, which is the standard method for accurate diagnosis. However, blood sampling is an invasive procedure that often causes patient discomfort, especially for individuals with needle phobia or vulnerable groups such as children and the elderly^9,10^. These procedures can be costly, requiring laboratory resources and trained personnel, which increases overall healthcare expenses and delays diagnosis by necessitating time-consuming sample processing and analysis, particularly when immediate intervention is critical. Furthermore, rural communities often face significant challenges in access to healthcare services, such as limited resources and a lack of equipment in medical facilities, resulting in poorer health outcomes for individuals in these areas. Early detection and timely treatment are crucial, but invasive diagnostic methods often delay diagnosis, especially in resource-limited areas. During the evaluation of a patient with suspected anemia, some observable physical examination/ophthalmological signs have been proven to correlate with the presence of the condition. For example, conjunctiva pallor is used as a preliminary observable indicator of anemia, allowing physicians to quickly estimate the Hgb deficiency without the need for immediate invasive testing^11,12^. Additionally, the redness of scleral blood vessels and its blueish tonality have also been recognized as potential signs of iron deficiency, as noted in recent studies^13,14^. Earlier noninvasive studies using digital conjunctiva images laid key groundwork for smartphone- or camera-based screening. In 2016, Collings et al.^15^ quantified a conjunctival erythema index (EI) from calibrated photographs and showed that palpebral conjunctival EI correlates with hemoglobin; using a compact camera and a smartphone, EI achieved clinically meaningful sensitivity/specificity and outperformed clinician assessment on internal validation, highlighting the feasibility of low-cost screening from consumer devices.

Digital image processing (DIP) and artificial intelligence (AI) have been increasingly integrated into clinical decision-support systems across multiple medical domains^16^, demonstrating their potential for early disease detection and efficient diagnosis. In oncology, for example, soft-computing and hybrid approaches have been applied to optimize feature selection, segmentation, and classification for cancer diagnosis, achieving improvements in accuracy and computational efficiency^17–20^. In ophthalmology, AI-driven pipelines have been extensively developed for glaucoma detection, with contributions ranging from automated type identification using machine learning (ML) and deep learning (DL)^21–24^, to optical coherence tomography-based smart systems for early recognition^25^. Beyond disease-specific applications, comprehensive analytical reviews have examined the strengths and limitations of machine learning algorithms in healthcare^26,27^ and have also explored broader trends in AI adoption in medicine^28^. On the other hand, specifically the ML and DL techniques, are research areas that have merged with medical areas^29^ and have equipped physicians with powerful tools for diagnosing, detecting, and analyzing various medical conditions. These approaches allow a precise analysis of visual markers and patterns, offering the potential for their application in both clinical and remote settings, thereby enhancing access to healthcare and improving diagnostic accuracy. From traditional DIP techniques to Convolutional Neural Networks (CNNs)^30^, these technologies have unlocked new possibilities for rapid and accurate solutions across multiple medical fields, including radiology, ophthalmology, oncology, and neurology, among many others^31^. Specifically for anemia detection, early techniques focused on detecting or segmenting red blood cells in microscopic blood samples to identify diseased sickle cells, a hematological disorder associated with anemia. For instance, the study carried out in 2014 by Elsalamony’s et al.^32^ demonstrated effective segmentation of these cells using Hough Transform combined with Neural Networks and Decision Trees. Following the analysis of microscopy image analysis, Nithya and Nirmala^33^ designed a framework in 2022, incorporating classical DIP techniques applied to blood smear images for later red blood cell counting and anemia screening. In more recent advancements in 2023, Appiahene et al.^34^ employed different ensemble models to analyze palm images of children for anemia prediction. Similarly, Asare et al.^35^ presented an analysis using classical ML classifiers for anemia screening using images of various body parts (palm, fingernails, and conjunctiva) for anemia screening, while Mahmud et al.^36^ focused on lip mucosa images and clinical Hgb levels to compare different ML classifiers and achieve high accuracy. In the particular domain of palpebral and conjunctiva images, several studies^37,38^ have reported high accuracy using diverse approaches, ranging from classical DIP approaches^39^ and ML classifiers^40,41^, Artificial Neural Network (ANN)^42^, Principal Component Analysis (PCA) and the K-Nearest Neighbor (K-NN)^43^, Local binary pattern (LBP) and Support vector machine (SVM)^44^, through recent CNN models^45–47^ till some hybridizations^48^. Complementarily, Kasiviswanathan et al.^49^ developed a U-Net-based semantic segmentation model for robust delineation of the conjunctiva under unconstrained imaging, explicitly targeting noninvasive anemia detection workflows and motivating segmentation-aware pipelines. These systems recognize subtle variations in different medical images that may indicate and estimate Hgb level or glaucoma screening, improving the accuracy and speed of early detection. However, these studies present a lack of explainability regarding their decision-making processes, making them a “black box” AI model, which represents a challenge to understanding and trusting the results of these models. These specific techniques will be further discussed and compared in this paper.

Recently, a novel AI approach called transformer^50^ has emerged as a tool with promising results in several areas^51,52^. Originally developed for natural language processing tasks^53^, the transformer architecture has been adapted to image analysis through models like the Vision Transformer (ViT)^54^, showing remarkable performance in various image classification tasks, including the medical area^55^. Unlike CNNs, which rely on local receptive fields to extract features from images, transformers use a self-attention mechanism that captures global relationships between different parts of the image. This enables transformers to perform well in scenarios where understanding the contextual relationship between distant image regions is critical for accurate diagnosis. Moreover, the integration of transformer-based models with explainable AI (XAI) techniques has made these systems more interpretable, intending to allow physicians to trust and understand the decision-making process of AI systems. While ViTs have been applied in other medical imaging domains, to the best of our knowledge, this is the first study exploring their use for non-invasive anemia detection from sclera-conjunctiva images. As described in the above paragraph, prior works in this area relied primarily on CNNs, classical image processing, or handcrafted features.

In this work, an innovative approach for anemia detection using conjunctiva and sclera images is presented. This proposal is mainly based on the ViT model, achieving higher performance when compared to other ML and DL techniques. The ViT model’s ability to capture and focus on relevant features within the images allows it to distinguish between anemic and non-anemic conditions accurately. Furthermore, considering the critical importance of model interpretability in healthcare, the attention map explainability technique is incorporated into the ViT model to provide visual insights into the specific regions of the images that the model considers most indicative of anemia. This enhances the transparency of the model’s decision-making process, making it more understandable for clinicians, hence facilitating its adoption in clinical practice. This proposal aims to significantly enhance access to healthcare, particularly in areas where traditional laboratory diagnostics may not be available, or scenarios where several patients have to be evaluated in a short time.

The main contributions of this paper are summarized as follows:

- A high-performance approach for diagnosing iron-deficiency anemia using images of the conjunctiva and sclera is introduced, demonstrating an alternative robust detection method without the use of invasive blood tests.
- An application of Vision Transformers (ViT) in analyzing medical images for anemia detection, proving its effectiveness for broader applications.
- The use of the attention map as an explainability method along with the ViT model to provide transparency in the model’s decision-making process.
- A comparison of state-of-the-art ML classifiers, CNNs architectures, and the ViT model for anemia screening, advancing in the assessment of up-to-date methods.

The contribution is therefore not the ViT architecture per se, but its tailored application, rigorous benchmarking against both CNN and ML baselines, and the integration of attention-based explainability to highlight clinically relevant cues.

## Materials and methods

This section provides an overview of the ViT architecture and the attention map visualizations used in the study. It also includes a dataset description, explaining the criteria for class separation based on clinical patient information. The data augmentation techniques employed to enhance the dataset are also discussed. Finally, the experimental setup and conditions are explained, along with a brief description of the techniques used for comparative analysis.

### Vision transformer (ViT)

The ViT architecture, introduced by Dosovitskiy et al.^54^, adapts the Bidirectional Encoder Representations from Transformers (BERT) model for image processing tasks. It works by splitting images into small patches, which are flattened and passed through a linear layer after adding positional encodings to preserve spatial information. For classification tasks, an additional token is introduced. These embedded patches are then processed by a transformer-based encoder, which utilizes a multi-head attention mechanism.

**Fig. 1:**
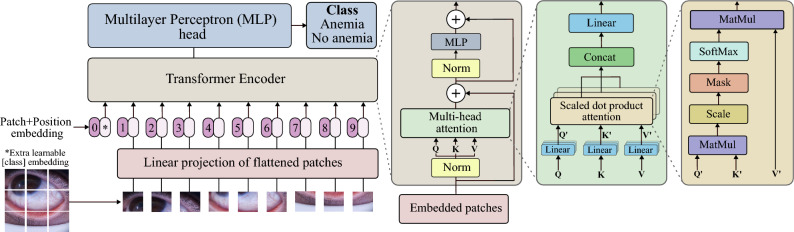
General scheme of the Vision Transformer (ViT).

Figure 1 illustrates the general flow of the ViT architecture. It depicts the division of images into patches, the addition of positional encodings, and the use of a transformer-based encoder with multi-head attention, emphasizing the role of Scaled dot product attention in capturing global relationships within the image.

The ViT starts by dividing an input image of size 224x224 pixels into fixed-size patches of 16$\times$16 pixels. Each patch is flattened and passed through a linear projection to form patch embeddings. A learnable position embedding is added to each patch embedding to retain spatial information. These patch embeddings, with position information, are fed into a standard transformer encoder. The encoder consists of multiple layers of self-attention and feed-forward neural networks. After processing through the transformer layers, the output corresponding to the [class] token (a special token for classification) is passed to a Multilayer Perceptron (MLP) head, which produces the final classification output.

At the core of this attention mechanism is the Scaled dot product attention, as proposed by Vaswani et al.^50^. In this mechanism, the Query ($\textbf{Q}$), Key ($\textbf{K}$), and Value ($\textbf{V}$) matrices are generated by applying linear transformations to the embedded patches. The $\textbf{Q}$ matrix is multiplied by the transposed $\textbf{K}$ matrix, and the result is scaled to stabilize gradients during training. Optionally, a mask can be applied at this stage. The resulting values are then passed through a SoftMax function, converting them into attention weights, which dictate the focus each patch receives. These attention weights are applied to the $\textbf{V}$ matrix, and the weighted values are then concatenated and passed through a linear projection to move to the next stage of processing.

In addition, the transfer learning (TL) approach can be applied within this framework by fine-tuning the pre-trained ViT model for specific tasks, such as anemia detection. In this process, the ViT model, pre-trained on large datasets like ImageNet^56^, is adapted by replacing its final classification layer. Depending on the task, the rest of the model can either be frozen or fine-tuned. This approach allows for faster convergence and improved performance when working with limited data, making ViT particularly effective in specialized applications.

#### Attention maps in ViT

One way to provide transparency into the features observed by the ViT model is through attention maps. These maps, generated by the Self-Attention (SA) mechanism, visualize how the model allocates its attention across different parts of the image. SA is essential for capturing long-range dependencies between elements in a sequence, making it a powerful tool in Transformer models. For 2D images, the SA mechanism operates on a sequence of flattened image patches. Specifically, an image $\textbf{x} \in \mathbb {R}^{H \times W \times C}$ is reshaped into a sequence of patches $\textbf{x}_p \in \mathbb {R}^{N \times (P^2C)}$, where *H*, *W*, and *C* denote the image’s height, width, and number of channels, respectively, and $N = \frac{HW}{P^2}$ represents the number of patches of size $P \times P$.

The SA mechanism calculates an attention score for each patch based on the inner product of the corresponding $\textbf{Q}$, $\textbf{K}$, and $\textbf{V}$ vectors. These vectors are learned through weight matrices $\textbf{W}^Q \in \mathbb {R}^{d \times d_q}$, $\textbf{W}^K \in \mathbb {R}^{d \times d_k}$, and $\textbf{W}^V \in \mathbb {R}^{d \times d_v}$, which project the input patches $\textbf{X}$ into the respective query, key, and value spaces. The attention matrix $\textbf{A} \in \mathbb {R}^{N \times N}$ is computed as:

1 $$\begin{aligned} \textbf{A} = \text {softmax}\left( \frac{\textbf{QK}^\top }{\sqrt{d_k}} \right) \end{aligned}$$

where $d_k$ represents the dimensionality of the key vectors, used to scale the dot product to avoid large values that could saturate the softmax function and lead to vanishing gradients. The output of the SA layer is:

2 $$\begin{aligned} \textbf{Z} = \textbf{A} \textbf{V} \end{aligned}$$

where $\textbf{V} = \textbf{X}\textbf{W}^V$, and $\textbf{W}^V$ represents the value matrix.

ViT utilizes Multi-Head Attention (MHA) to enhance the model’s ability to focus on various aspects of an image simultaneously. In MHA, multiple heads operate in parallel, each with its own set of query, key, and value projections. Each head captures distinct features or patterns from the image, leading to a more comprehensive understanding. The output of MHA is obtained by concatenating the results from all heads:

3 $$\begin{aligned} \text {MHA}(\textbf{Q}, \textbf{K}, \textbf{V}) = [\textbf{Z}_0, \textbf{Z}_1, \dots , \textbf{Z}_{h-1}] \textbf{W}^O \end{aligned}$$

where $\textbf{W}^O \in \mathbb {R}^{h \times d_v \times N}$ is a learnable weight matrix that linearly combines the outputs from the *h* heads.

Visualizing these attention maps provides valuable insights into how the model prioritizes different regions of an image when making predictions. The aggregation of attention maps from multiple heads offers a nuanced and multifaceted view of the image, revealing which regions are most influential in the model’s decision-making process.

### Dataset

The Eyes-defy-anemia dataset^46,57,58^ was specifically designed for estimating anemia diagnosis based on conjunctival pallor. It is composed of 218 images acquired using a smartphone, a magnifying lens, and a 3D-printed support. The dataset includes external views of the eye, showing both the sclera and conjunctiva zones, as will be shown in the Results and analysis section. The dataset comprises images from Italian and Indian patients, with 123 and 95 images, respectively, and is accompanied by the Hgb levels at the time of acquisition. However, the dataset does not provide an anemia/no-anemia classification; therefore, all class labels in this study were assigned directly from the clinical hemoglobin measurements included in the dataset, obtained through standard blood tests. These labels reflect each patient’s confirmed hematological status rather than any visual assessment, and one sample lacking Hgb information was excluded to maintain clinical validity.

Because pregnancy status was unavailable, sex-specific thresholds were applied with a screening orientation. For the Indian subset, an Hgb < $12\,\textrm{g}/\textrm{dL}$ threshold was used for women, while an Hgb < $14\,\textrm{g}/\textrm{dL}$ threshold was used for men. The $12\,\textrm{g}/\textrm{dL}$ cutoff for non-pregnant women follows WHO guidance; the often-cited 11 g/dL threshold applies to pregnancy^59,60^, which cannot be ascertained here. For men, $14\,\textrm{g}/\textrm{dL}$ is consistent with recent imaging-based screening studies (e.g., non-contrast CT) that prioritize sensitivity when flagging suspected anemia for confirmatory testing^61,62^. For the Italian patient set, a threshold of Hgb $<10.5$ $\textrm{g}/\textrm{dL}$ is used, as determined by the dataset authors in previous studies^46,63^, since no gender information was provided for this set. The dataset includes some demographic and acquisition variability, but not a fully representative global sample. As mentioned above, all images were captured with a smartphone camera, a magnifying lens, and a 3D-printed support, which introduces minor differences in angle and lighting, but does not cover the broad variability of devices, nor the broad ethnic representation that may occur in clinical practice. These characteristics should be considered when interpreting the reported performance and underscore the need for validation on larger, multi-center, and more diverse cohorts. These characteristics should be considered when interpreting the reported performance.

**Fig. 2:**
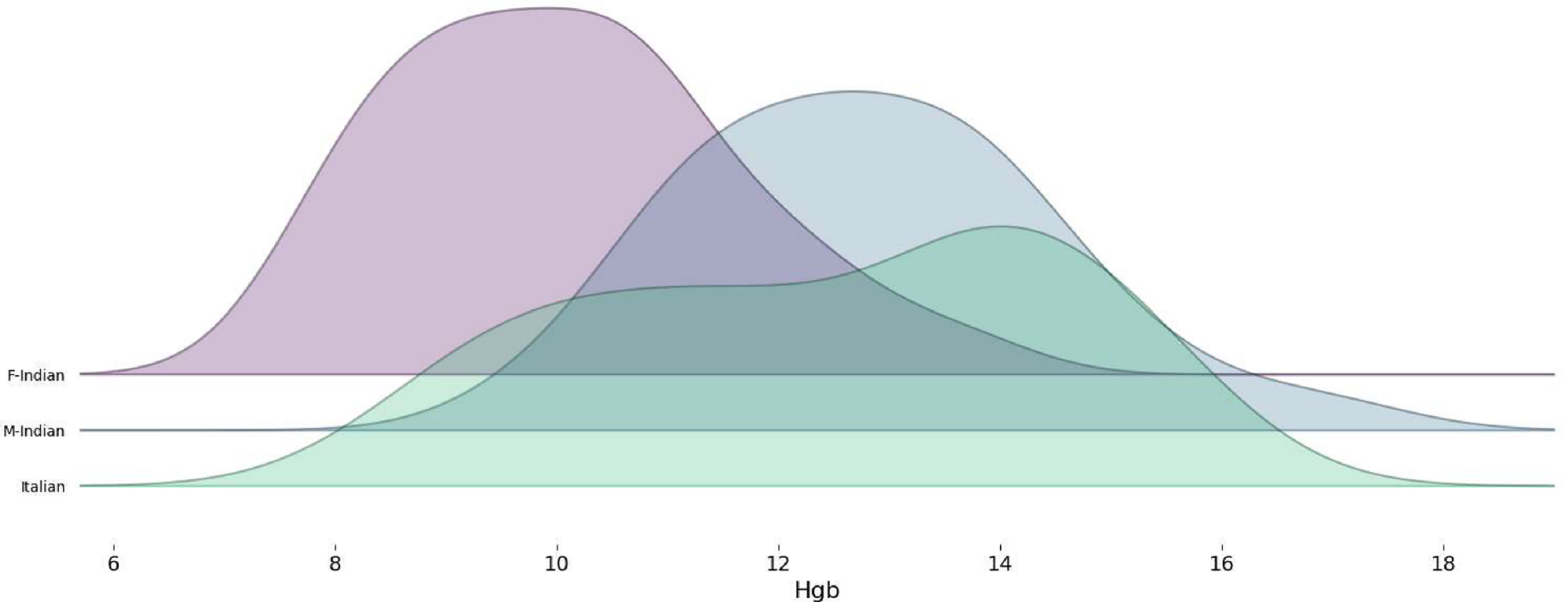
Class density distribution of Indian and Italian sets.

As mentioned above, one image of an Italian patient was removed from this dataset since no Hgb level was provided. The Hgb density distribution in each set is presented in Fig. 2.

Considering the Hgb level distribution over both Italian and Indian image sets and the Hgb level criteria described above, all images were mixed to generate a single broader dataset with a total of 217 images, split into 126 and 91 for “No anemia” and “Anemia” classes, respectively.

#### Data augmentation

The dataset used for the training process consists of raw and augmented images categorized into “No anemia” and “Anemia” classes. The classified dataset using the above-described criterion initially contained 127 images for “No anemia” and 91 images for “Anemia”.

To improve generalization and reduce overfitting on this relatively small dataset, simple yet clinically appropriate augmentation was implemented. Specifically, horizontal and vertical mirroring were applied to all images. This strategy increases sample diversity by simulating left/right eye orientations and variations in patient positioning during image capture, while preserving the diagnostic features of conjunctiva pallor, scleral hue, and vascular visibility. Color jitter, brightness scaling, or elastic deformations were intentionally avoided, as these could artificially distort the subtle chromatic and structural markers of anemia, thereby improving robustness without altering medically relevant features. Such conservative augmentation strategies are widely adopted in medical imaging tasks with limited datasets, as they enhance model stability while safeguarding the clinical interpretability of the input images^64–66^. After augmentation, the dataset was expanded to 378 images for “No anemia” and 273 images for “Anemia”, as presented in Table 1.

**Table 1:** Dataset distribution for raw and augmented images.

**Category**	**No anemia**	**Anemia**	**Total**
Raw images	126	91	217
Augmented data	378	273	651

The Italian and Indian cohorts were first combined into a single dataset, after which horizontal and vertical flips were applied to all raw images. These conservative transformations preserve the diagnostic chromatic and structural cues associated with conjunctival pallor and scleral appearance, and therefore do not introduce new label-relevant information. Because augmentation was performed before the 80/20 split, both training and testing subsets contain only orientation variants of the original images. Although such a strategy may raise concerns about data leakage, the flips used here do not generate artificial features, alter hemoglobin-related cues, or reveal information unavailable in the raw images. The merged cohort and conservative augmentation thus maintain the validity of the subsequent train-test evaluation.

It is important to acknowledge that the dataset used in this study is relatively small. Additionally, the two subsets (Italian and Indian patients) are imbalanced in size, and only the Indian subset provides gender information. The absence of sex data in the Italian cohort necessitated the use of a uniform Hb threshold, which may limit the precision of classification.

### Experimental configuration

To numerically compare the performance of the ViT in this task ML algorithms and CNN-based models were employed using the same augmented dataset for classification, namely, SVM^67^, Naïve Bayes^68^, and XGBoost^69^ for ML analysis, and Inception-V3^70^, DenseNet161^71^, MobileNet-V2^72^, and ResNet-50^73^ for DL comparison. All the CNNs and ViT models were trained using the same hyperparameters for over 30 epochs with a batch size of 8, while the loss was computed using the Cross-Entropy Loss^74^ for binary classes. Regarding the data split, an 80-20 approach was implemented for training and testing, respectively. For the optimization stage, the Nesterov accelerated Adaptive Moment Estimation (Nadam)^75^ is used. It was configured with a learning rate of $1 \times 10^{-5}$ and a weight decay parameter of $1 \times 10^{-4}$ to reduce overfitting.

The ViT used in this study corresponds to the ViT-B/16 architecture (google/vit-base-patch16-224-in21k), which operates on 16$\times$16 image patches and includes 12 transformer encoder layers, each with 12 self-attention heads and a hidden embedding dimension of 768. The model contains approximately 86 million parameters.

To reduce the training computational cost using the TL approach, pre-trained weights were loaded to all CNNs using the *ImageNet-1K*^76^ dataset approach, and input image size as defined in the original paper of each model. On the other hand, the ViT model was trained with no TL, and with both its pre-trained versions on the *ImageNet-1K* and *ImageNet-21k*^77^ datasets using a $224\times 224$ pixels image size. For the SVM, Naive Bayes, and XGBoost training and testing performance, the same batch size and image resize of the ViT were applied.

To ensure a fair comparison, all CNNs and ViT variants were trained and evaluated under the same data split and hyperparameters (batch size, optimizer, learning rate, weight decay, and epochs), as detailed above. Although an 80/20 train-test split was used for all models, the limited dataset size poses inherent overfitting risks. To mitigate these risks, a conservative augmentation strategy (horizontal and vertical flips only) and closely monitored training and validation curves were applied for early signs of divergence. More aggressive augmentation was intentionally avoided to preserve clinically meaningful chromatic cues and prevent distortions of the subtle features associated with conjunctival pallor and scleral coloration.

All experiments were performed on a workstation with an AMD Ryzen 5 2600X CPU, 32 GB of RAM, and an NVIDIA RTX 2060 GPU (6 GB VRAM). Training the ViT-B/16 model for 30 epochs required approximately 30 minutes, while the CNN baselines required between 30 and 50 minutes, depending on model complexity, with MobileNet-V2 being the fastest and DenseNet-161 the slowest due to parameter count.

### Classification performance metrics

In this approach, different metrics are employed to assess the effectiveness of the tested methods in accurately classifying the anemia image. In the evaluation of algorithms, there are four key cases to consider: the number of positive cases correctly predicted as positive ($\text {TP}$), the number of negative cases correctly predicted as negative ($\text {TN}$), the number of positive cases incorrectly predicted as negative ($\text {FN}$), and the number of negative cases incorrectly predicted as positive ($\text {FP}$).

From those measurements, different metrics are derived to analyze the classification performance, namely Accuracy, Precision, Recall, and *F*1-score, whose calculation is defined below^78^. Accuracy measures the proportion of correct predictions relative to the total number of cases in the evaluated set. On the other hand, Precision measures the proportion of true positive predictions against the total number of correct predictions made, measuring the algorithm’s ability to predict positive cases. Recall, also called the true positive rate (TPR), shows the proportion of true positives between the sum of true positives and false negatives (the real number of positive cases). Finally, *F*1-score is defined as the harmonic mean of precision and recall; the two metrics are combined in one expression to measure the trade-off between precision and recall. This metric is useful when there is a class imbalance. The calculation of all metrics is presented in Eqs. 4 through 7.

4 $$\begin{aligned} & \text {Accuracy} = \frac{\text {TP} + \text {TN}}{\text {TP} + \text {TN} + \text {FP} + \text {FN}} \end{aligned}$$

5 $$\begin{aligned} & \text {Precision} = \frac{\text {TP}}{\text {TP} + \text {TN}} \end{aligned}$$

6 $$\begin{aligned} & \text {Recall} = \frac{\text {TP}}{\text {TP} + \text {FN}} \end{aligned}$$

7 $$\begin{aligned} & F1\text {-score} = 2\times \frac{\text {Precision}\times \text {Recall}}{\text {Precision}+\text {Recall}} \end{aligned}$$

## Results and analysis

This section presents the classification numerical results of all tested models and an analysis of the obtained attention maps obtained through the ViT, to analyze its explainability capabilities for further discussion.

### Classification results

The results in Table 2 show a clear distinction between the models in terms of all performance metrics. Notably, the ViT model with *ImageNet-21k* TL achieves the best performance across all metrics, with an impressive $98.47\%$ overall accuracy. This model also achieves near-perfect precision, recall, and F1-score for both Anemia and No Anemia, indicating its robustness in classification tasks. The ViT model highlights the positive effect of TL. ViT achieves an accuracy of $89.31\%$ without pre-trained weights, but with *ImageNet-1k* TL, it jumps to $95.42\%$, results higher than any other CNN loading the pre-trained weights of the same dataset. It is important to note that every instance classified as anemia by the ViT model with *ImageNet-21k* is correct, with no false positives as denoted with perfect precision, while correctly identifying all cases of no anemia, achieving perfect recall with no false negatives.

Compared with the best CNN baseline (MobileNet-V2, 94.66% accuracy), ViT with ImageNet-21k TL improves overall accuracy by +3.81 percentage points while maintaining perfect precision for Anemia and perfect recall for No Anemia on the test set. This, together with the consistent gains from no-TL to 1k-TL to 21k-TL, underscores the importance of transfer learning for this task. The dataset labels themselves were derived from clinical Hgb measurements, meaning that the reported performance reflects agreement with the invasive diagnostic gold standard.

**Table 2:** Performance metrics for Anemia and No Anemia classification.

**Model**	**Anemia**			**No anemia**			**Overall accuracy (%)**
	**Precision**	**Recall**	**F1-score**	**Precision**	**Recall**	**F1-score**	
**SVM**	0.64	0.69	0.66	0.84	0.82	0.83	77.86
**Naïve Bayes**	0.79	0.83	0.81	0.83	0.79	0.81	80.92
**XGBoost**	0.85	0.81	0.83	0.89	0.91	0.90	87.79
**Inception-V3**	0.92	0.88	0.90	0.88	0.92	0.90	90.08
**DenseNet-161**	0.92	0.90	0.91	0.94	0.95	0.95	93.13
**ResNet-50**	0.92	0.95	0.93	0.96	0.93	0.94	93.89
**MobileNet-V2**	0.90	0.96	0.93	0.97	0.94	0.6	94.66
**ViT (no TL)**	0.83	0.95	0.89	0.95	0.85	0.90	89.31
**ViT (with** ***1k* TL)**	0.90	0.98	0.94	0.99	0.94	0.96	95.42
**ViT (with** ***21k* TL)**	**1.00**	**0.97**	**0.98**	**0.97**	**1.00**	**0.99**	**98.47**

**Fig. 3:**
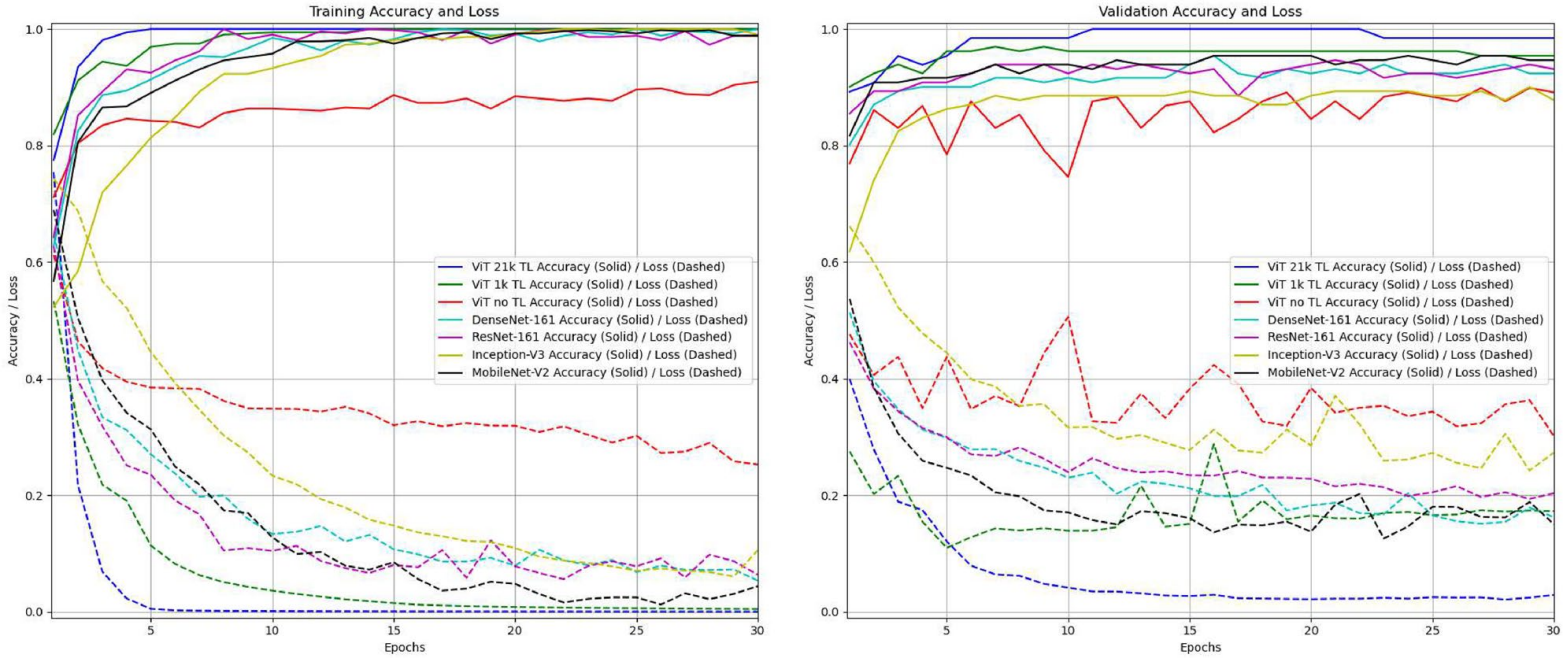
Training and validation curves for accuracy and loss of ViT and tested CNNs.

In Fig. 3 the training and validation curves for accuracy and loss of ViT and tested CNNs are presented. In general, the ViT model pre-trained on the larger dataset (ViT *21k* TL) consistently achieves the highest accuracy and lowest loss in both training and validation. This demonstrates the significant benefit of transfer learning for generalization. These results demonstrate that the ViT models, especially with large-scale TL, significantly outperform traditional CNN models and other ML approaches, making them the most effective for Anemia classification. Its ability to focus on the relevant visual regions, coupled with classification performance metrics, demonstrates its potential for high-precision medical imaging applications.

**Fig. 4:**
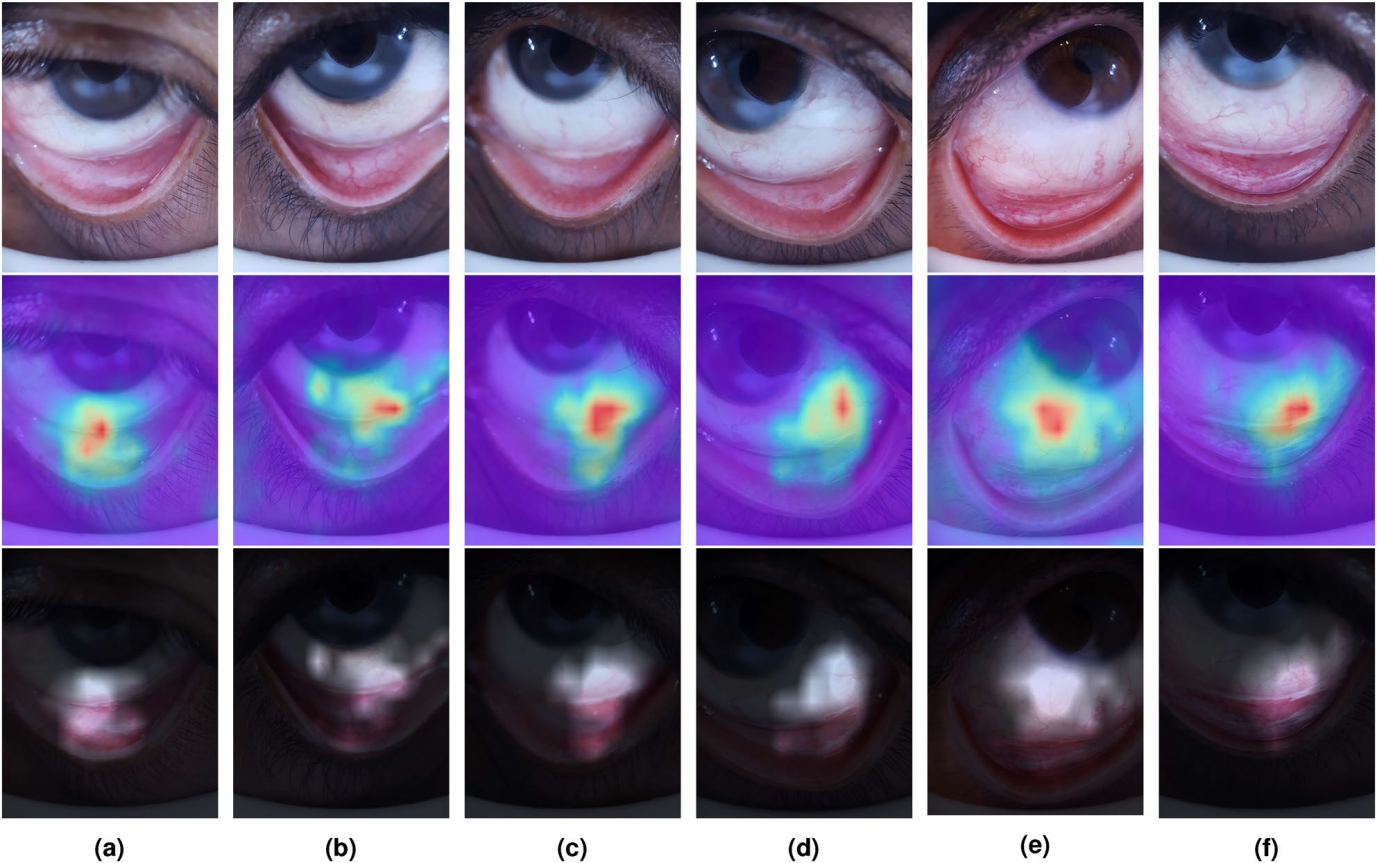
Examples of attention visualizations generated by the ViT model. Each column (**a**)-(**f**) corresponds to one case, with the first image showing the raw input, the second the attention heatmap overlay, and the third the transparency visualization. Columns (**a**)-(**c**) illustrate anemia cases, while (**d**)-(**f**) illustrate no anemia. Highlighted regions correspond to clinically meaningful cues such as conjunctival pallor, scleral hue, and vascular patterns.

The ViT architecture is particularly suitable for sclera-conjunctival analysis because its self-attention mechanism captures global relationships between distant patches, enabling the model to integrate distributed cues such as conjunctival pallor, scleral hue, and vascular prominence that are not confined to local neighborhoods.

To complement the aggregate metrics, Table 3 summarizes the results on the held-out test set for the best-performing ViT model (*ImageNet-21k* TL). The dataset contained 273 anemia and 378 no-anemia samples, of which 20% (55 anemia, 76 no-anemia) were reserved for testing.

**Table 3:** Confusion matrix of the best ViT (*ImageNet-21k* TL) on the test set.

	**Predicted anemia**	**Predicted no anemia**
Actual anemia	53	2
Actual no anemia	0	78

As shown, the ViT model achieved perfect precision for no-anemia (no false positives) and near-perfect recall for anemia, with only a small number of false negatives. Out of 55 anemia cases in the test set, 53 were correctly identified (TP), while only 2 were missed (FN). Importantly, all 78 no-anemia cases were correctly classified (TN), with no FP. This outcome reflects the model’s perfect precision for the anemia class, ensuring that individuals flagged as anemic by the model truly had the condition. At the same time, the near-perfect recall indicates that almost all anemic patients were detected, with only a marginal number overlooked. Clinically, this profile is desirable because it minimizes the risk of unnecessary interventions for healthy individuals, while maintaining a very high probability of detecting anemia cases that require follow-up.

Full k-fold cross-validation was not performed due to the small and demographically heterogeneous nature of the dataset (Italian and Indian cohorts with different hemoglobin thresholding criteria), which limits the statistical value of repeated folding. The consistent performance across the ViT-no-TL, ViT-1k-TL, and ViT-21k-TL configurations already provides indirect evidence against severe overfitting, as progressively richer pretraining priors stably improve accuracy. In addition, formal statistical significance testing was not conducted because each model was trained as a single deterministic run using a fixed train-test split, leaving no repeated measurements from which to estimate variance. Under these conditions, traditional inferential statistics (e.g., t-test or ANOVA) are not applicable. The substantial and consistent performance gap between ViT-B/16 and the CNN baselines (8–20 percentage points, depending on architecture) further reduces the likelihood that the improvements are incidental.

### Explainability of decisions

In this work, explainability is provided through attention map visualizations derived directly from the ViT’s multi-head self-attention mechanism. Unlike post-hoc methods such as Grad-CAM, these attention maps reflect the model’s native decision process by identifying which image patches receive the highest attention during classification.

In Fig. 4, examples of attention maps generated by the ViT model are displayed. The first row shows the raw images, the second one presents the attention heatmap superimposed on the respective raw image to highlight the relevance of each patch as determined by the ViT, and the third row illustrates the attention map with transparency overlaid on the raw images, providing a clearer visual emphasis most relevant to the model.

These results demonstrate the effectiveness of this proposal by accurately highlighting key regions of interest. Despite the palpebral and conjunctival regions not being specifically labeled for the ViT model, the attention heatmap predominantly focuses on the inner conjunctiva or the lower eyelid. For example, the fifth column attention map finely delimits the iris/sclera separation, proving the patch-wise analysis efficiency. The transparency visualization further confirms that the model’s attention aligns with these critical areas, reinforcing the interpretability and reliability of the model’s predictions. This visualization also helps ensure that the ViT model is not centering its attention on irrelevant parts of the image. This alignment with clinical assessment practices suggests that ViT not only performs well in terms of screening but also provides interpretable results that correspond to visual elements considered by physicians. This interpretability is crucial for validating model predictions and ensuring that the model’s focus aligns with expert diagnostic practices.

Previous approaches primarily focused on segmenting and classifying palpebral regions in this dataset, neglecting the sclera as a significant diagnostic area. However, attention map visualizations in this study demonstrated that the sclera plays a critical role in enhancing diagnostic accuracy. This finding challenges prior assumptions and shows that incorporating the sclera as a relevant zone can improve diagnostic performance in anemia detection.

**Fig. 5:**
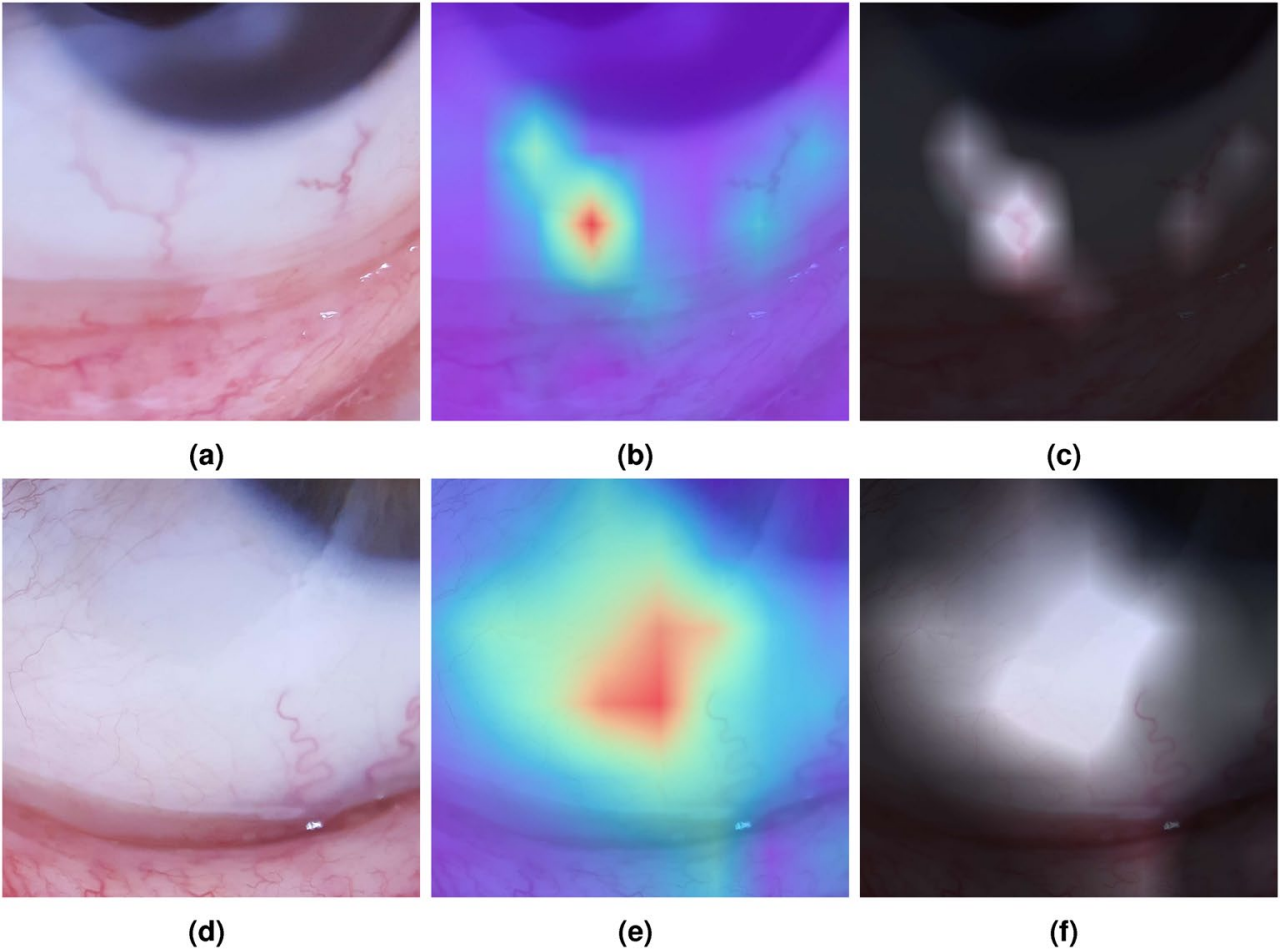
Zoomed-in relevant attention zones. (**a**) and (**d**) shows the raw input image, (**b**) and (**e**) its respective attention heatmap, and (**c**) and (**f**) the transparency of each image.

Figure 5 shows zoomed-in raw, attention, and transparency heat maps of two images to further analyze the attention map behavior. As observed in the corresponding heatmaps (b) and (e), attention is focused on relevant features such as the vessels and pallor areas which are clinically relevant markers of anemia. The overlaid transparency maps (c) and (f) further emphasize these regions, demonstrating the model’s capacity to identify significant features like vascular structures and plain scleral coloration. This meticulous analysis provides insight into the model’s decision-making process, offering a clearer understanding of the model’s diagnostic reasoning.

Attention maps consistently emphasized clinically relevant regions (inner conjunctiva, lower eyelid, sclera/iris boundary, vascular structures), supporting the hypothesis that ViT’s attention helps capture subtle chromatic and textural indicators of anemia while offering transparent visual explanations of the decision process. It is noteworthy that the regions emphasized by the attention maps are clinically recognized markers for anemia^13–15^. Clinically, the attention consistently focuses on zones routinely examined by clinicians when visually assessing potential anemia. This alignment between model attention and clinical diagnostic cues enhances interpretability and supports practitioner trust in the model’s decisions. This overlap suggests that the model’s visual explanations are not arbitrary but align with established medical reasoning. While the present work did not include a formal validation with ophthalmologists or hematologists, an informal review by a clinical collaborator acknowledged in this paper confirmed that the highlighted regions correspond to expected diagnostic cues.

While the attention map visualizations provide intuitive and anatomically meaningful insights, the present work does not include a quantitative evaluation of explainability because the dataset lacks region-level clinical annotations (e.g., conjunctival boundaries, scleral segmentation, or expert-marked diagnostic ROIs). As a result, attention maps could only be assessed qualitatively. Nonetheless, the highlighted regions consistently overlapped with areas routinely examined by clinicians, as confirmed through previously mentioned informal medical feedback.

Nonetheless, several limitations must be acknowledged. First, the dataset is restricted to Italian and Indian cohorts, which may not fully capture ethnic, demographic, and acquisition variability. The proposed ViT-based framework has not yet been tested on external or real-world datasets outside the Eyes-defy-anemia collection. As no additional publicly available conjunctiva-sclera datasets with clinical hemoglobin ground truth currently exist, the present work should be interpreted as a proof-of-concept demonstration. Second, validation on larger and more diverse, multi-center cohorts is needed to ensure the model s robustness and generalizability across different populations, devices, and imaging conditions.

Despite the promising performance, several limitations must be acknowledged. The dataset, although specifically curated for anemia detection, remains relatively small and restricted to Italian and Indian patients, introducing potential demographic and acquisition-related biases. While conservative augmentation mitigates overfitting, broader generalizability must be demonstrated through validation on larger, multi-center datasets encompassing diverse populations, imaging devices, and acquisition conditions. Practical deployment also presents challenges, including variability in smartphone or camera quality, inconsistent lighting during image capture, and the need for seamless integration into existing healthcare workflows. The smartphone-based acquisition design nevertheless makes the approach compatible with point-of-care or mobile health platforms, where a lightweight application could capture a standardized ocular image and provide a preliminary anemia risk indicator prior to laboratory testing. To move from proof-of-concept to clinical implementation, regulatory requirements must be met, including adherence to Software as a Medical Device (SaMD) guidelines^79^, multi-center clinical validation studies, documentation of performance across demographic groups, and the adoption of privacy-preserving strategies. Together, these considerations highlight both the promise of this methodology and the necessary steps for safe and equitable clinical deployment.

### State-of-the-art comparison

As mentioned, several proposals using palpebral and/or conjunctival images have been presented in recent years. In Table 4 a comparison of the ViT against methods used for detection in similar imaging is presented. As can be observed, traditional methods such as color thresholding, clustering, and linear models (e.g., K-means, PCA, and multiple regression) achieved moderate accuracy levels ranging between $78.9\%$ and $90.00\%$; particularly, the XGBoost reached a notable $93.00\%$. These methods mainly rely on basic image segmentation and feature extraction techniques, limiting their ability to capture more complex patterns in conjunctiva and palpebral images. On the other hand, more recent methods utilizing CNNs and hybrid techniques have significantly improved performance, achieving accuracies in the range of $91.00\%$ to $93.7\%$. These DL approaches have enhanced feature extraction capabilities, enabling them to capture richer image details.

**Table 4:** Performance metrics of state-of-the-art techniques for anemia screening in conjunctiva/palpebral images classification.

**Author**	**Year**	**Main technique**	**Accuracy (%)**
Tamir et al.^39^	2017	Color thresholding	78.90
Sevani et al.^40^	2018	K-means clustering	90.00
Jain et al.^42^	2020	ANN	97.00
Asiyah et al.^43^	2022	PCA and K-NN	87.50
Appiahene et al.^37^	2023	CNN + Logistic regression	92.50
Dimauro et al.^46^	2023	MobileNetV2	91.00
Purwanti et al.^45^	2023	ResNet-50	93.70
Bhusham et al.^47^	2023	DenseNet-201	93.70
Sehar et al.^38^	2024	Multiple regression model	83.30
Priyadarshini et al.^41^	2024	XGBoost	93.00
Mythily et al.^44^	2024	LBP + SVM	93.00
Muljono et al.^48^	2024	SVM + MobileNetV2	93.00
**ViT (with** ***1k* TL)**	2024	ViT	95.42
**ViT (with** ***21k* TL)**	2024	ViT	**98.47**

In contrast, the ViT model, achieving an accuracy of $95.42\%$ and $98.47\%$ using *ImageNet-1k* and *ImageNet-21k* TL approaches, respectively, significantly outperforms all state-of-the-art methods. This notable improvement is likely due to the ViT’s ability to process images in a patch-wise manner, capturing more global dependencies in the image, including those in the scleral zone, which was often neglected in earlier segmentation-based models. The attention maps generated by ViT have shown that relevant information for anemia detection is distributed not only in the conjunctiva but also in the sclera, suggesting the importance of analyzing this broader region for improved diagnostic accuracy.

## Conclusions

This work proved the effectiveness of using ViT for non-invasive anemia detection through images of the conjunctiva and sclera. The ViT model, enhanced by the TL approach, outperformed traditional ML models and CNN architectures, achieving a $98.47\%$ overall accuracy and emphasizing the importance of explainability in medical AI applications.

When compared to state-of-the-art proposals, this approach surpasses them in performance but also offers a significant advancement in explainability terms. Instead of processing the entire image as a whole, ViT divides the image into smaller patches, which allows the model to capture global dependencies and contextual information across the image more effectively than CNNs, which in turn enhances explainability. Each patch can be independently analyzed to understand its contribution to the model’s prediction.

By using attention maps as an explainability technique, this approach offers a transparent decision-making process, allowing physicians to understand how and why the model reaches its conclusions. These maps highlight key regions of the conjunctiva and sclera that are clinically associated with anemia without any prior knowledge of the clinical relevance of these areas. This reinforces the clinical significance of these zones in visual analysis, as the model autonomously identifies and emphasizes the regions that align with established medical understanding, validating its diagnostic relevance.

Although the ViT with transfer learning achieved high accuracy, these results should be interpreted with caution. The current dataset is relatively small, geographically limited (encompassing only Italian and Indian cohorts), and lacks essential metadata, such as sex, in one subset, thereby restricting its demographic representativeness. Uniform acquisition conditions (smartphone with magnifying lens) may further reduce the variability typically encountered in clinical practice, raising the risk of overfitting. Accordingly, future work must validate the model on larger, multi-center datasets, incorporating stratified multi-fold evaluation once more balanced and ethnically diverse cohorts become available, collected under variable acquisition conditions and complemented by fairness audits to ensure equitable performance across demographic groups, thereby enabling a more comprehensive assessment of robustness and generalizability.

In terms of deployment, the smartphone-based acquisition protocol and lightweight ViT architecture make this approach conceptually suitable for point-of-care or field screening, particularly in low-resource settings. Nevertheless, real-world deployment will require standardized image acquisition procedures, privacy-preserving implementation (e.g., on-device or institutionally hosted inference with appropriate encryption), and formal regulatory and ethical approval, in addition to multi-center external validation.

Regulatory approval will demand prospective multi-center trials, risk assessments, and demonstration of safety and efficacy. While these results highlight the potential of lightweight ViT architectures with transfer learning for real-time point-of-care anemia screening in low-resource environments, the approach should be viewed as an augmentation tool rather than a replacement for invasive diagnostics. Promising future directions include federated learning for privacy-preserving multi-institutional training and systematic expert validation to assess the alignment of AI explanations with clinical judgment quantitatively. Together, these steps will be essential to move this work from proof-of-concept toward safe and reliable real-world deployment.

However, this study demonstrates that ViT models are a promising alternative for non-invasive anemia screening, providing both high screening performance and interpretability, a key requirement for integrating medical AI systems into clinical practice.

## Data Availability

The dataset analysed during the current study is available in the following IEEE DataPort repository: https://ieee-dataport.org/documents/eyes-defy-anemia.

## Code availability

The source code of this proposal can be found at https://github.com/Oscar-RamosS/ViT-Anemia-Detection.
